# Supplementary material for: Identification and functional analysis of non-coding regulatory small RNA FenSr3 in Bacillus amyloliquefaciens LPB-18
Source: PeerJ. 2023 May 15;11:e15236. doi: 10.7717/peerj.15236 (PMC10194069; doi:10.7717/peerj.15236)
Supplement: Supplemental Information 4 [file peerj-11-15236-s004.zip › KO/CK-vs-T1_map/map04660.html]

KEGG PATHWAY: T cell receptor signaling pathway - Reference pathway


|  |  |
| --- | --- |
| **T cell receptor signaling pathway - Reference pathway** |  |

[
Pathway menu
| Organism menu
| Pathway entry
| Show description
| User data mapping
]

Activation of T lymphocytes is a key event for an efficient response of the immune system. It requires the involvement of the T-cell receptor (TCR) as well as costimulatory molecules such as CD28. Engagement of these receptors through the interaction with a foreign antigen associated with major histocompatibility complex molecules and CD28 counter-receptors B7.1/B7.2, respectively, results in a series of signaling cascades. These cascades comprise an array of protein-tyrosine kinases, phosphatases, GTP-binding proteins and adaptor proteins that regulate generic and specialised functions, leading to T-cell proliferation, cytokine production and differentiation into effector cells.

|  |  |  |
| --- | --- | --- |
| Reference pathway | 184% 150% 122% 100% 82% 67% 55% | 图片下载 |
